# European Aluminum Group (Aluminum in Biological Systems)

**DOI:** 10.1155/MBD.1995.247

**Published:** 1995


					EUROPEAN.AL,UM!NUM GROUP
(Aluminum B olog cal Systems)
(Analytical Chemistry, Physiology, Toxicology in vivo and in vitro,
Inorganic Chemist, Modelling, Spectroscopy)
Aluminum, the most abundant metal ion in the earth's crust, is involved in important
pathologies (aluminopathies) related to uremic subjects in dialysis treatment. In
addition, aluminum could be a risk factor to subjects who receive parenteral
nutrition and pharmacological treatment. Particularly exposed appear to be the
infants. Aluminum has been also hypothesized to be involved, most likely as a
secondary factor, in Alzheimer's disease.
The data bank BIOSIS shows that, every year, about 1200 scientific publications
are produced by the scientific community around the world on different aspects of
aluminum studies.
Since 1985, we have constituted in Padova, Italy (University and National Research
Council) an interdisciplinary group formed by chemists, biologists, medical doctors,
pharmacologists, dealing with various aspects of aluminum in biological systems
with the aim to better understand the role of this metal in analytical chemistry,
physiology and nutrition, toxicology, pharmacology and pathology.
With the aim to enlarge an exchange of information, we wish to propose the
formation of an European Aluminum Group to share information, publications and
to organize workshops as well as to promote more interest in this metal at
conferences on Metal Ions in Biological Systems.
All European and Non-European colleagues interested to join this group can
contact
Paolo Zatta
Center for the Study of Physiology and Biochemistry of Metalloproteins
University of Padova
Department of Biology
Via Trieste, 75
35131 Padova, Italy
E-mail" zatta @cribil.bio.unipd.it
Fax: + 30 49 828 6359
Tel." 39 49 828 6361
and send him their name, address, fax and telephone numbers, and e-mail address
as well as their field of interest, research specialization and titles of three of their
most relevant papers.
247

				

